# 

**Published:** 1875-05

**Authors:** 


### THE AMERICAN JOURNAL OF DENTAL SCIENCE,

EDITED BY

F. J. S. GORGAS, M. D., D. D. S.

VOL. 9.—THIRD SERIES.

S.G.O. LIBRARY

BALTIMORE:

SNOWDEN & COWMAN.

LONDON:

TRUBNER & CO., 60 PATERNOSTER ROW.

1875—1876.

### INDEX TO VOLUME IX.---THIRD SERIES.

A Biographical Sketch of Life of Dr. T. B. Hamlin, 72
A Case of Reflex Neuralgia, - - - - 44
A Dentist in the Newspapers, - - - 68
A Fundamental Requisite, - - - - 35
A New Cause for Blue Line on the Gums, - - 237
A New Splint for Fractured Jaw, - - - 228
A Convenient Local Anodyne, - - - 192
A Sad Occurrence, - - - - - 183
A Medical Imbroglio, - - - - 136
A New Method of applying the Rubber Dam, - 37
A New Motor, - - - - - 176
A Protest, - - - - - - 43
A Cure for Drunkenness, - - - 240
A Calculous of Stenos Duct, - - - 239
A Dangerous Compound, - - - - 239
A New Mode of Burial, - - - - 237
A New Pair of Siamese Twins, - - - 573
A British Dental Association, - - - 239
Aconite as a Local Anæsthetic, - - - 26
Address, - - - - - 498, 529
Action of Mercury upon the Blood, - - - 189
Adulteration of Beeswax, - - - - 334
Alumni Meeting, - - - - - 558
Amalgams, - - - - - 134
Ambler, H. L., M. D., D. D. S., - - - 562
American Dental Association, 118, 260, 311, 361, 404, 458
American Academy of Dental Science, - 167, 460
American Academy of Dental Surgery, Proceedings of,
233, 285, 337, 385, 505, 552
American Dental Convention, - - - 79, 117
An Improved Method of Taking Plaster Impressions, 76
An Easy Method of Removing Part of Inferior Dental Nerve, 181
An Indigestible Morsel, - - - - 48

Antidote to Chloroform, - - - - - - 94
A Large Mouth, - - - - - - 240
A Few Facts from Salter, - - - - 268
A Reply, - - - - - - 379
A Photographic Wonder, - - - - 335
A Strange Freak, - - - - - - 427
A Tooth in the Tongue, - - - - - 140
An Advance in Dental Surgery, - - - 240
An Excellent Tooth Wash, - - - - 525
Allen, Paul W., M. D., - - - - 26
Alternation a Law of Vital Action, - - - 289
Alumni Meeting, - - - - - - 470
Antidote to Carbolic Acid, - - - - 474
Artificial Teeth on Protruding Gums, - - - 21
Application to Burns, - - - - - 478
Are Irregularities of the Teeth ever Caused by Tongue and Thumb Sucking, - - - - - 455
Arsenious Acid, Physiological Action of, - - 87
Atkins, Francis H., M. D., - - - - - 228
Barnes, H. J., M. D., - - - - 217
Barrett, Dr. C. W., - - - - - 538
Base for Teeth, New, - - - - - - 38
Baltimore College of Dental Surgery, - - 39, 561

BIBLIOGRAPHICAL.

A Case of Reflex Neuralgia, - - - - 44
A Protest, - - - - - - - 43
Fifth Report New York Ophthalmic and Aural Institute, - - - - - - 44
Reflex Irritations of the Genito-urinary Tract, - 44
The Chemists and Druggists Diary for 1875, - 43
Transactions of N. Y. Odontological Society, - 43
The Sanitarian, - - - - - 44, 431
The Pen and Plow, - - - - - - 44
The Herald of Health, - - - - 44
The Histology and Histochemistry of Man, - 285
The Cholera Epidemic of 1873 in U. S., - 286
Transactions of College of Physicians of Philadelphia, 286
Transactions of Medical and Chirurgical Faculty of Maryland, - - - - - - 286

Transactions of American Med. Association for 1875, 429
The American Agriculturist, - - - 430
Circular of Information, - - - 430
Dentists' Appointment Book and Daily Record, 431
The Physicians Diary for 1876, - - 431
The Herald of Health, - - - - 432
Questions on the Structure and Development of the Human Teeth, - - - - 429
Vernon Galbray, or the Empiric, - - 429
Vick's Floral Guide for 1876, - - - 430
Billroth on the Repair of Tissues, - - - 186
Bicarbonate of Soda for Toothache, - - 526
Black, G. V., D. D. S., - - - - 8, 93, 241
Boehm, Prof., M. D., - - - - 87
Borate of Soda Gargles, - - - - 476
Briggs, C. S., M. D., - - - - - 464
Buckingham, Chas. E., M. D., - - - 26
Building on a Part of the Crowns of Teeth, - - 4
Burkholder, N. M., D. D. S., - - - 445
Burns and Scalds, Treatment of, - - - 23
California State Dental Association, - - 80
Campbell, James, M. D., - - - - 17
Camphor Poisoning, - - - - 528
Camphorated Chloral in Neuralgia, - - - 187
Cancer of the Mouth, Local Treatment of, - 33
Cancer of Tongue and Jaw, - - - - 526
Carbolic Acid as an Antiphlositic, - - - 236
Case of Abstention from Food. - - - 144
Causes of Irregularity in the Development of the Teeth, 145, 193
Celluloid, - - - - - - 404
Celluloid, Notes on, - - - - 1, 49
Celluloid, The Right to Use, - - - 42
Celluloid Manufacturing Co., - - - - 138
Celluloid, Does the Use of, Infringe the Cummings Patent, 425
Celluloid Case, Important Decision in the, - - 461
Chase, Henry S., D. D. S., M. D., - - - - 167
Chloral for Ulcers, - - - - - 479
Chloral Hydrate, External Use of, - - - 336
Chloride of Lead as a Deodorizer and Disinfectant, - 524

Chloroform Asphyxia, How to Prevent, - - 571
Cobb, S. J., D. D. S., - - - 62, 72, 303
Cold Powder, - - - - - 142
Collodion in Treatment of Carious Teeth, - - 527
Consanguineous Marriages, - - - 188
Correction, - - - - - - 379
Croton Chloral Hydrate in Neuralgia, - - 432
Curious Brain Lesions, - - - - 383
Cushing, Geo. H., D. D. S., - - - 63, 97
Davis, Dr. R. B., - - - - - - 548
Dawson, B. F., M., D., - - - - 174
Death from Chloroform, - - - - 525
Death from Ether Inhalation, - - 235, 527, 573
Death from Nitra-venous Injection of Chloral, - 96
Death while Unconscious from Ether, - - - 379
Death from Chloroform, - - - - 45
Death from Methylene, - - - - - 46
Deciduous Teeth, Treatment of - - - 167
Dental Fees, Prosecution for, - - - - 52
Dental Education, - - - 89, 303
Dental Profession in France, - - - - 179
Dentistry, The Progress of - - - . - 281
Detection of Falsification of Wax, - - - 46
Deleterious Effects from Artificial Teeth on Rubber Base, 129
Dental Neuralgia, - - - - - 384
Dental Education, - - - - - - 567
Dental Pulp, its Pathology and Treatment, - - 395
Dental Legislation, - - - - - 538
Diagnosis of the Lead Line, - - - - 142
Driscoll, W. E., M. D., - - - - 21
Dispensing with Rain, - - - - - 287
Diseases Ascribed to Dentition and their Pathological Admissibility, - - - - - 382
Dislocating the Jaw for Chloroform Narcosis, - 525
Dying on the Operating Table, - - - - 427
Eastlake, W. C., D. D. S., - - - - - 354
Eczema from Tincture of Arnica, - - - - 572
Effects of Inhalation of Chloroform during Parturition on the Fœtus, - - - - - 288

Efficiency of Mineral Water in Reducing and Adding Fat, 237
Epistaxis Arrested by Compressing the Facial Arteries, 189
Ether, Death from - - - - - - 379
Ether vs. Chloroform, - - - - - 288
Ether Spray as an Anæsthetic, - - - - 471
Exposed Pulps, Treatment of - - 221, 276, 467
Exposed Pulps, - - - - - 324
Exhaustive Effects of Dental Practice, - - - 566
External Use of Chloral Hydrate, - - - - 336
Filling Teeth, - - - - - - 515
Filling Teeth with Tin, - - - - - - 562
Food for Infants During Dentition, - - - - 174
Georgia State Dental Society, - - - - - 164
Glass Cement, - - - - - - 480
Glycosuria, - - - - - - 477
Gold, - - - - - - - - 8
Gold Screws for Retaining Fillings, - - - 548
Gunning, T. B., D. D. S., M. D. - - - - 5
Ham, S. F., M. D., - - - - - 37
Hemorrhagic Diathesis, - - - - 131
Hogden, John T., M. D. - - - - - 181
Hodgkin, Prof. Jas. B., D. D. S., - - 1, 49, 538
How far are we Justified in Anticipation Proximate Decay, 210
How to Remove Stains from Steel Instruments, - 575
Hunter, E. L., D. D. S., - - - - 4
Illinois State Dental Association, - - - 7
Ingersoll, L. C., M. D., - - - - - - 289
Intra-venous Injection of Chloral for Anæsthesia, - 96
Irregularity in the Development of the Teeth, - 145, 193
Kentucky State Dental Association, - - - 8
Kingsley, Norman W., D. D. S., - - - - 145, 193
Lancing the Gums, - - - - - - - 26
Lead Poisoning in Lead Workers, - - - - 143
Letter from * * * * - - - - 6
Letter from Dr. Nash, - - - - - 135
Letter from W. J. Kellett, - - - - - 521
Lewis, Theo. G., D. D. S., - - - - - 225
Line, J. Edw., D. D. S., - - - - 268
Location of the Sense of Taste, - - - - 236

Making a New Nose out of a Finger, - - 477
Massachusetts Dental Society, - - - - 119
McQuillen, J. H., M. D., D. D. S., - - - 455
Mechanical Dentistry, - - - - - 250
Medical and Dental Graduates, - - - 188
Memoir of Dr. Geo. Sidney Jones, - - - 163
Medical Diploma for Women, - - - 528
Mississippi State Dental Association, - - - 79
Moffatt, Geo. T., M. D. - - - - 460
Mollier, M. - - - - - - 327
Mode of Administering Ether, - - - 478
Monument in Memory of Dr. Horace Wells, - - 84
Morgan, W. H., D. D. S., M. D., - - - 163
Morris, John, M. D., - - - - - 23
Morphia in Neuralgia, - - - - 570
Narcotizing Horses, - - - - - 191
Nelaton's Method of Resuscitation, - - - 94
Neuralgia Treated by Nerve Stretching, - - 384
New Base for Teeth, - - - - - 38
New Method of Arresting Hemorrhage, - - - 190
New Remedy for Burns, - - - - 477
Nicholls, J. E., M. D., - - - - - 33
Nitrous Oxide in Luberculosis, - - - 192
Nitrous Oxide Gas, - - - - - 217
OBITUARY.
Dr. M. McCarty, - - - - 62
Dr. O. N. Allan, - - - - - 141
Dr. R. M. Gage, - - - - 233
Oleate of Mercury for Ringworm, - - - 48
On Filling Teeth, - - - - - 575
On the Preservation of the Deciduous Teeth, - - 445
On Right Handedness, - - - - 45
On the Physiological Action of Arsenious Acid, - 87
Osteo-Sarcoma of the Lower Jaw, - - - 464
Over Work or Over Play, - - - - 238
Partial Excision of Upper Jaw for Enchrondroma, - 126
Peabody, D. D., M. D., - - - - - 467
Perine, Geo H., D. D. S., - - - 515
Physiological Experiment upon the Human Corpse, - 236

Plastic Operation for the Lower Lip, - - - 475
Poisoning by Carbolic Acid, - - - - - 47
Poisoning by Aconite, - - - - - 235
Prevention and Treatment of Decay on Proximal Surfaces of Teeth, - - - - - - 97
Probabilities, - - - - - - 241
Prosecution for Dental Fees, - - - - 52
Pyæmia from Alveolar Abscess, - - - 346
Quinine Gargle in Sore Throat, - - - - 476
Relation of Alcohol to Animal Heat and Muscular Power, 192
Relation of Imperfect Teeth to Cataract, - - - 31
Relief of Pain from External use of Chloral, - - 48
Relief of Tooth-Ache by Bicarbonate of Soda, - 384
Salicylic Acid, - - - - - - 82, 120
Salicylic Acid as an Antiseptic, - - - 330
Salicylic and Benzoic Acids, Antiseptic Properties of - 480
Salicylic Acid, A New Solvent for, - - - 571
Salivary Fistula, Radical Cure of - - - 568
Singular Death from Ether, - - - - 95
Solubility of Salicylic Acid, - - - 190
Solvents for Rubber, - - - - 95, 574
Southern Dental Association, - - - 471
South Carolina State Dental Association, - - 116
Stevens, Mordaunt, M. R., C. S., D. D. S. - - - 179
Summer Sessions, - - - - - - 182
Susquehanna Dental Association, - - - 7, 333
Structure of Tumors as Related to their Character, - 191
Snow as a Hæmostatic, - - - - 192
Swain, E. D., D. D. S., - - - - - - 210
Subcutaneous Injection of Water, - - - - 524
Suggestions, - - - - - - - 354
Syphilis of Parents Affecting the Offspring, - 383
Teeth and Toothache, - - - - - 347
Teeth and Oatmeal, - - - - - - 47
Thackston, W. W. H., M. D., D. D. S., - - 498, 529
The Eye Tooth and the Eye, - - - - 17
The Seventh District Dental Society of N. Y. - - 80
The Murder of Two Dentists, - - - - 183
The Teeth as Sexual Organs, - - - - - 188

The Late Meeting at Long Branch, - - - - 231
The Anti-Neuralgic Properties of Essence of Mint, - 336
Third Dentition, - - - - - - 382
Third Dentition at the Age of Seventy-Three, - - 575
Thompson, James F., D. D. S., M. D., - - 455
Thymol, as an Antiseptic, - - - - 143
Toothache, - - - - - - 236, 575
Treatment of Neuralgia, - - - - 473
Treatment of Colds, - - - - - 93
Treatment of Facial Neuralgia, - - - 474
Treatment of Diptheria, - - - - 575
Treatment of Exposed Pulps, - - 221, 276, 467
Treatment of a Fractured Tooth, - - - 225
To the Dental Profession, - - - - 523
Turner, J. Smith, M. R., C. S., - - - 76
Tumors of the Tongue, - - - - - 327
Virginia State Dental Association, - 379, 433, 481
Warts, - - - - - 192, 527
Welchens, Samuel, D. D. S., - - - 221, 276
What may our Patients Reasonably Expect shall be Accomplished by Operations upon the Teeth, - 63
Where to Look for Arsenic in Cases of Poisoning, - 527
Willmott, J. B., D. D. S., - - - - 395
Willson, O., Dr., - - - - - 250
Wine Drinking and Drunkenness, - - - 237
Women Doctors in Russia, - - - - 191

### *Susquehanna Dental Association.*

The Annual Meeting of this Association will be held at Scranton, Pennsylvania, on Wednesday, May 12th, 1875, at 10 o'clock A. M. Session to continue to two days. Orders for Excursion Tickets ean be had by applying to

W. H. Hertz, D. D. S., *Rec. Secretary,*
Hazleton, Pa.

---

### *Illinois State Dental Association.*

The Eleventh Annual Meeting of this Association will convene at Ottawa, Illinois, on Tuesday, May 12th, 1875.

A cordial invitation to be present is extended to all progressive Dentists.

Charles E. Koch, D. D. S.,

*Secretary Ill. S. D. Association.*

*Kentucky State Dental Association.*

The Annual Meeting of this Association will commence on Tuesday, June 1st, 1875, at Lexington, Ky.

A cordial invitation is extended to all dental practitioners to be present.

DR. B. OSCAR DOYLE, *President.*
CHAS. E. DUNN, *Secretary.*

---

### SELECTED ARTICLES.

### TO READERS AND CORRESPONDENTS.

Communications intended for publication, and exchange journals and papers, should be addressed to the EDITOR OF THE AMERICAN JOURNAL OF DENTAL SCIENCE, 259 N. Eutaw St., Baltimore, Md. All letters relating to the business of the journal, or containing cash remittances, to be directed to Messrs. SNOWDEN & COWMAN. Articles for publication should be received by the editor at least three weeks previous to the first day of the month on which the number in which it is designed for them to appear, is issued.

### CONTENTS:

### ORIGINAL COMMUNICATIONS.

ARTICLE I.—Notes on Celluloid. By Prof. James B. Hodgkin, D. D. S. .... 1

ARTICLE II.—Building on a Part of the Crowns of Teeth. By E. L. Hunter, D. D. S. .... 4

ARTICLE III.—Letter from Dr. T. B. Gunning .... 5

ARTICLE IV.—Letter from * * * .... 6

Susquehanna State Dental Association .... 7

Illinois State Dental Association .... 7

Kentucky State Dental Association .... 8

### SELECTED ARTICLES.

### MONTHLY SUMMARY.

Death from Chloroform .... 45

On Right-Handedness .... 45

Detection of Falsification of Wax .... 46

Death from Methylene .... 46

Poisoning by Carbolic Acid .... 47

Teeth and Oatmeal .... 47

Relief of Pain by External Use of Chloral .... 48

Oleate of Mercury in Treatment of Ringworm .... 48

An Indigestible Morsel .... 48

☞ The American Journal of Dental Science will contain fifty pages of reading matter in each number, making a volume of not less than

*Six Hundred* pages.

THE

### AMERICAN JOURNAL
OF
### DENTAL SCIENCE.

F. J. S. GORGAS, M. D., D. D. S., Editor.

The *Journal* is issued on the first of each month and contains not less than *fifty* pages of reading matter in each number, exclusive of advertisements, making a volume of *six hundred* pages per annum.

In each number will be found Original Communications, Correspondence, Selected Articles, Monthly Summary, Bibliographical Notices and Editorial Matter, and every effort will be exerted to render the *Journal* worthy of the patronage of the Dental profession.

A generous co-operation is respectfully solicited from the members of the profession; and as the *Journal* has now a printing office of its own, which materially lessens the cost of its publication, it will be issued at the reduced subscription price of

**$2.50 Per Annum in Advance.**

Communications are respectfully solicited on all subjects pertaining to the practice of dentistry, as well as reports of cases occurring in dental practice.

### RATES OF ADVERTISING.

| | 1 MO. | 2 MO. | 3 MO. | 6 MO. | 12 MO. |
|---|---|---|---|---|---|
| 1 PAGE. | $15 00 | $22 50 | $30 00 | $50 00 | $100 00 |
| ½ " | 10 00 | 15 00 | 20 00 | 30 00 | 50 00 |
| ¼ " | 7 00 | 11 00 | 15 00 | 20 00 | 30 00 |
| ⅛ " | 5 00 | 8 00 | 11 00 | 15 00 | 20 00 |

All original communications designed for publication, works for review, new instruments for notice, exchanges, &c., should be directed to the editor, 259 N. Eutaw St., Baltimore, Md.

All advertisements and subscriptions should be addressed to

SNOWDEN & COWMAN,

*Publishers of the Am. Journal of Dental Science.*

86 W. FAYETTE ST. BET. CHARLES & LIBERTY STS.,

BALTIMORE, MD.

